# An Exceptionally Favorable Response to Etoposide and Cisplatin

**DOI:** 10.7759/cureus.418

**Published:** 2015-12-18

**Authors:** Gautam Valecha, Adarsh Vennepureddy, Uroosa Ibrahim, Marcel Odaimi

**Affiliations:** 1 Medicine, Staten Island University Hospital; 2 Hematology-Oncology, Staten Island University Hospital

**Keywords:** small cell lung carcinoma, response, survival, etoposide, cisplatin, sclc

## Abstract

A 66-year-old female with multiple medical co-morbidities was diagnosed with limited-stage small cell lung carcinoma (SCLC) about 11 years ago, back in 2004. The patient was treated with concomitant chemotherapy and radiotherapy, along with prophylactic whole brain radiation. She received a total of four cycles of etoposide and cisplatin. The patient showed a complete response to the above-mentioned treatment and had no evidence of tumor recurrence on any of the scans until 2015. Her last computed tomography (CT) scan of the chest in October 2015 showed bilateral hilar and mediastinal lymphadenopathy. Fine needle aspiration (FNA) of the left hilar node revealed the presence of malignant cells consistent with SCLC. Median survival for limited stage SCLC ranges from 16-24 months, and the reported five-year survival is 14%. In this report, we present the case of a 66-year-old female who showed an exceptionally favorable response to cisplatin and etoposide chemotherapy characterized by a disease-free survival of 11 years.

## Introduction

Small cell lung carcinoma (SCLC) accounts for approximately 15% of all bronchogenic carcinomas. Despite the advances in its treatment and the tumor's favorable response to both chemo and radiation therapy, the prognosis of this aggressive cancer still remains poor. The median survival for SCLC ranges from 16-24 months, and the reported five-year survival is about 14% [1]. We present the case of a 66-year-old female diagnosed with limited-stage disease (LD) SCLC in 2004. She was treated with chemotherapy and thoracic radiation therapy (TRT) along with prophylactic whole brain radiation. The patient showed an exceptionally favorable response to the treatment characterized by a disease-free survival of 11 years.

## Case presentation

Informed patient consent was obtained. No identifying patient information was disclosed in this paper.

**Figure 1:**
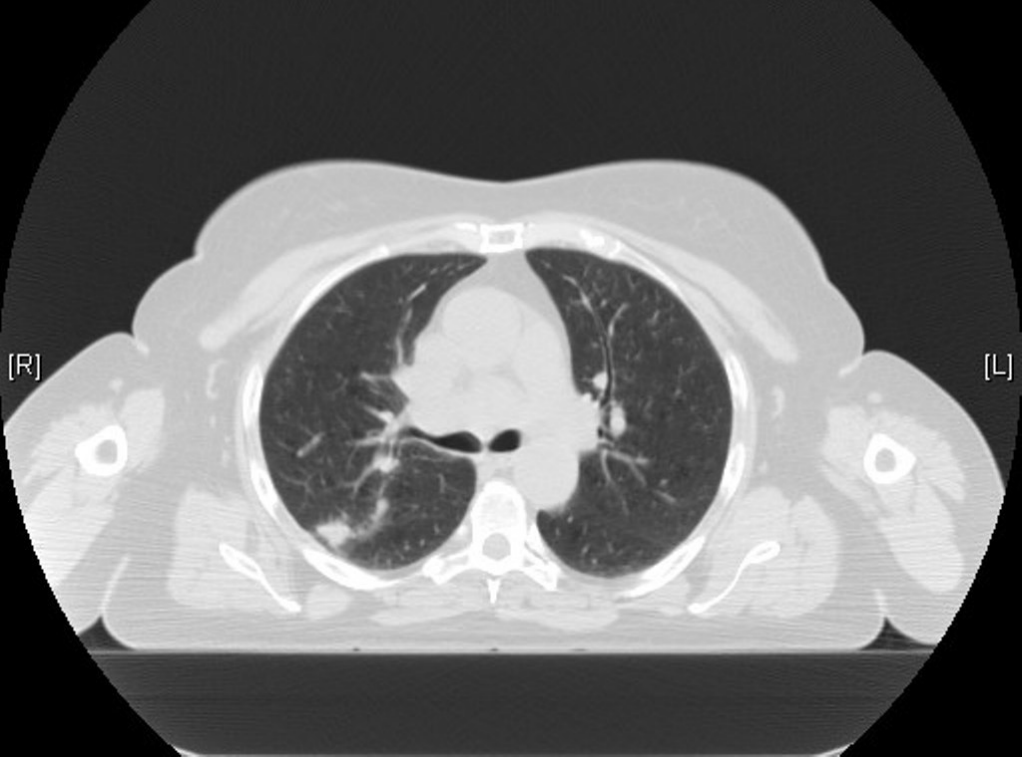
CT scan of the chest from 2004 demonstrating nodular opacity in the posterior aspect of right upper lobe

**Figure 2:**
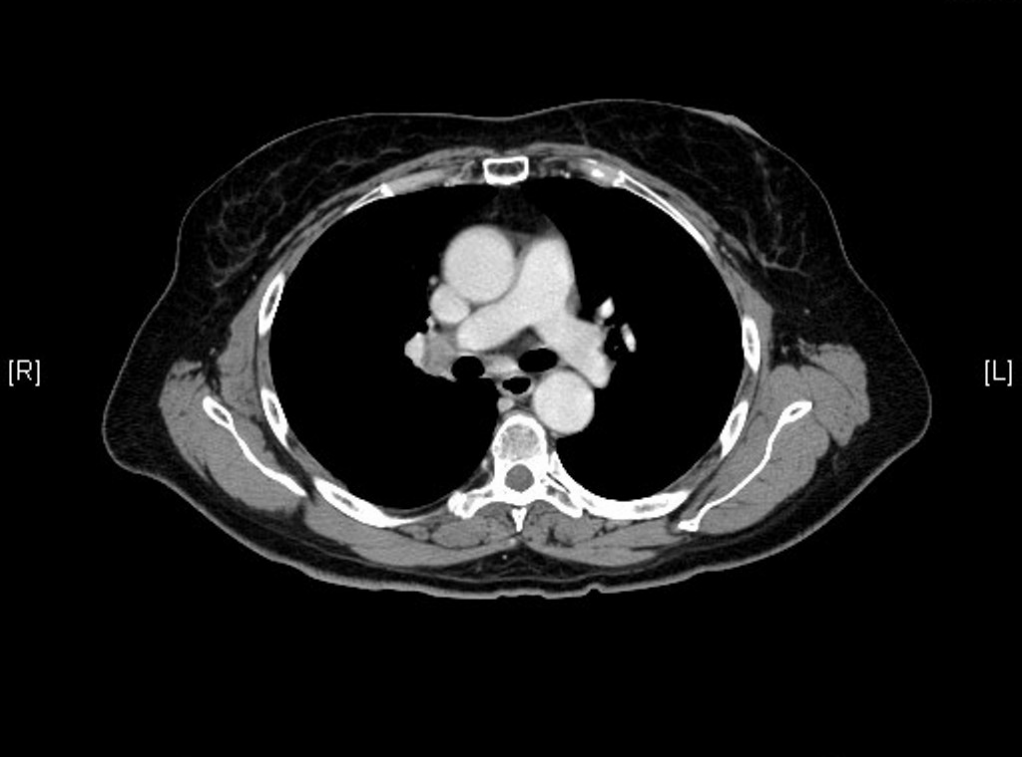
Hilar adenopathy seen on CT scan of the chest from 2004

A 66-year-old female with a medical history of hypertension, diabetes mellitus, dyslipidemia, osteoarthritis, hypothyroidism, emphysema, cholelithiasis, and surgical history of laparoscopic cholecystectomy and appendectomy was diagnosed with limited-stage SCLC in 2004. She also had a smoking history of 40 pack/years. The patient's initial computed tomography (CT) scan of the chest (Figures 1-2) showed a large 3 cm nodule in the posterior aspect of the upper lobe of the right lung, along with the extensive involvement of ipsilateral hilar and mediastinal lymph nodes. CT-guided fine needle aspiration (FNA) was used to establish the diagnosis of SCLC. No evidence of metastasis to other sites was noted on a CT scan of the abdomen-pelvis, magnetic resonance imaging (MRI) of the brain, bone scan, or positron emission tomography (PET) scan.

**Figure 3:**
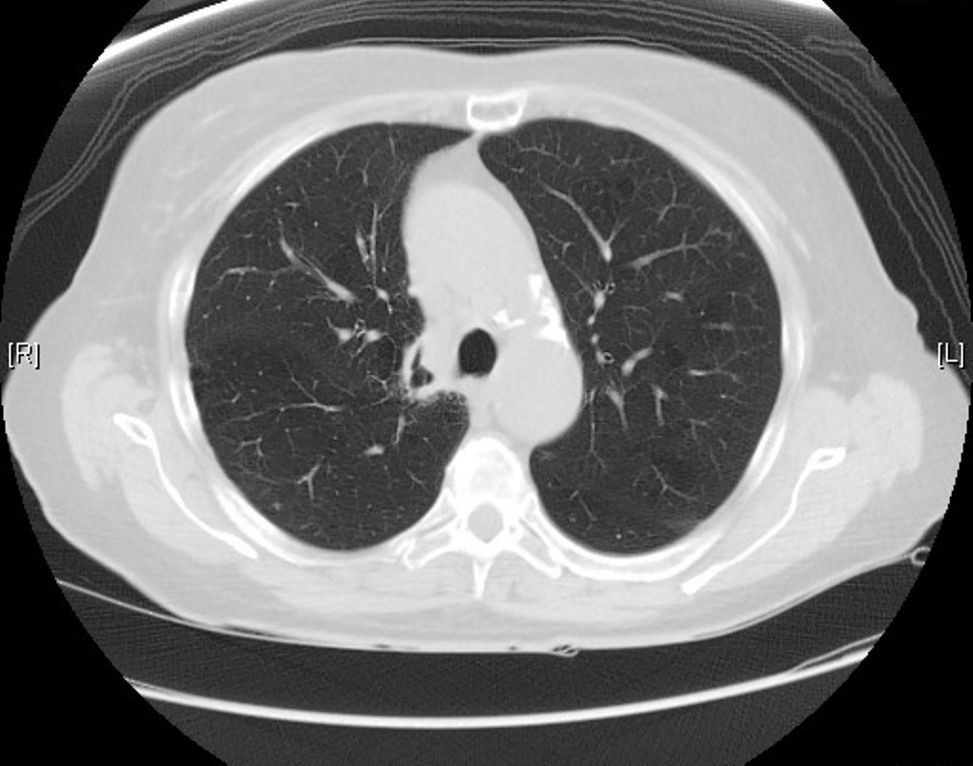
Post-chemotherapy CT scan of the chest from 2005 showing resolution of parenchymal nodular opacity

**Figure 4:**
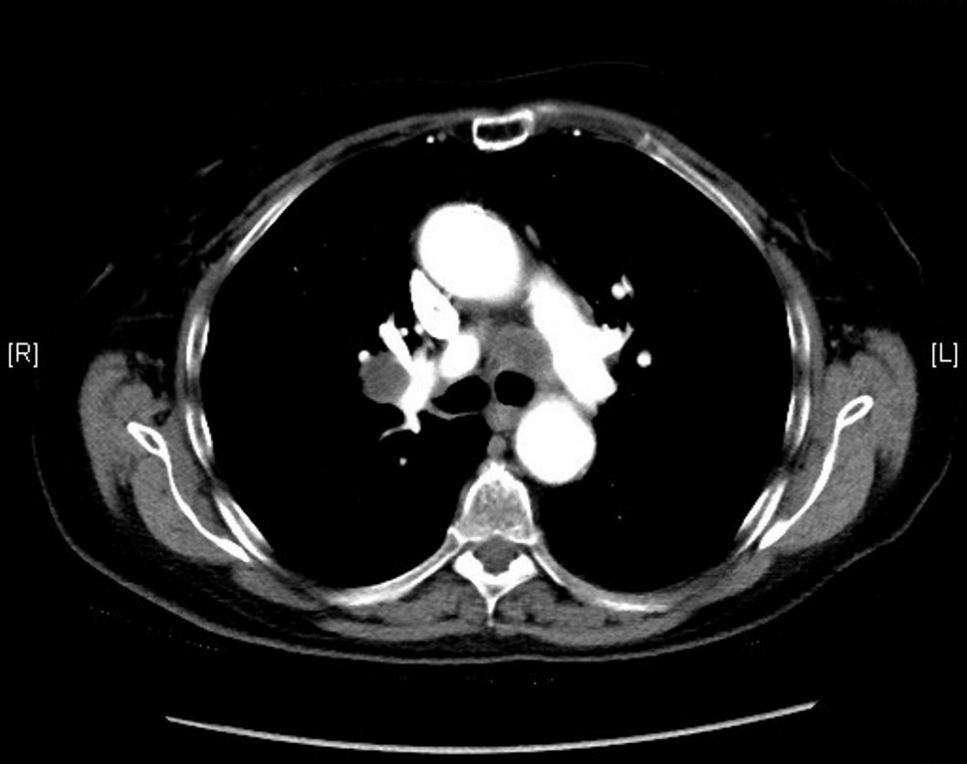
CT scan of the chest from 2015 showing precarinal adenopathy

**Figure 5:**
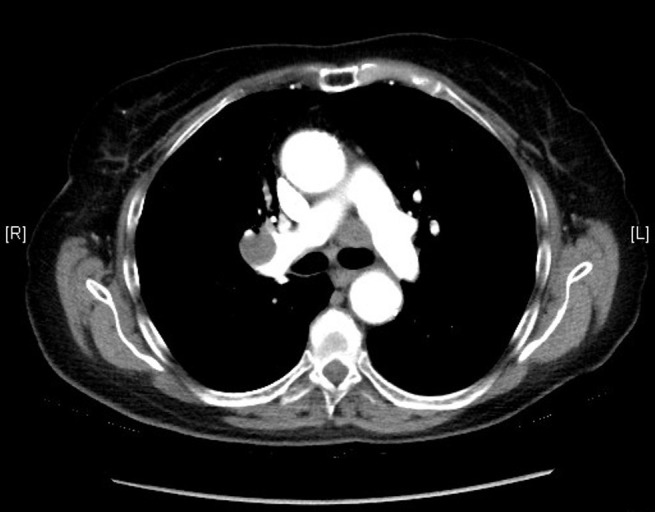
CT scan of the chest from 2015 showing right hilar adenopathy

The patient was started on etoposide and cisplatin therapy. She received a total of four cycles of etoposide and cisplatin (days 1, 2, and 3) every 21 days. Etoposide was administered at 100 mg/m^2 ^(total 160 mg) and cisplatin at 25 mg/m^2^ (total 40 mg) per dose. She also received concomitant radiation therapy for the primary tumor. In addition, she also underwent prophylactic whole brain radiation. After receiving the above-mentioned treatment, the patient was closely followed up with CT scans of the chest every six months until 2009 and then every year until 2014. No evidence of recurrence was seen on any of these scans (Figure 3). However, the last CT scan of the chest performed in October 2015 showed interval development of right hilar (2.8 x 1.9 cm), right prevascular (1.7 x 1.6 cm), subcarinal (1.4 x 1.1 cm), right paraesophageal (1.8 x 1.7 cm), left paratracheal (2.2 x 1.7 cm), and left subaortic (1.7 x 1.6 cm) adenopathy (Figures 4-5). The patient subsequently underwent an endobronchial ultrasound (EBUS) in November 2015. FNA of the left hilar lymph node revealed the presence of malignant cells consistent with SCLC. Immunohistochemical stains were positive for synaptophysin, focally positive for CD56 and chromogranin, and negative for CK7, CK20, TTF1, CDX2, PAX8, napsin, p63, CK5/6, and CD45. Currently, the patient is considering different treatment options, and a decision about her future course of treatment is yet to be taken.

## Discussion

SCLC accounts for about 15% of all bronchogenic carcinomas. It is an aggressive subtype of lung cancer characterized by early local, regional, and distant metastases. Without treatment, the median survival from the time of diagnosis is approximately two to four months [1]. In the United States, there will be approximately 221,200 new cases of lung cancer and over 158,040 deaths in 2015. However, the overall incidence and mortality of SCLC have decreased in the last few decades.  

Combined-modality treatment with etoposide-cisplatin and TRT is the most widely used treatment option for patients with LD SCLC. Clinical trials have consistently demonstrated median survivals of 18 to 24 months with less than 3% treatment-related mortality. Prophylactic cranial radiation in LD prevents central nervous system recurrence, may improve the long-term survival in patients with good response to chemoradiation, and offers palliation of symptoms in metastatic brain disease [1].

SCLC responds better to chemotherapy and radiation than all other subtypes of lung cancer. However, even with treatment, the median survival of LD is approximately 16 to 24 months, and five-year survival is about 14%. An analysis study was performed by Lassen, et al. on 1,714 unselected patients with SCLC treated with combination chemotherapy in nine consecutive clinical trials from 1973 to 1991. Of the 1,714 patients (828 with limited-stage disease (LD) and 886 with extensive-stage disease (ED)), only 60 patients survived for more than five years (40 with LD and 20 with ED). Late relapses occurred in 15% of the five-year survivors and secondary malignancies in 20%. The five-year survival rate was 3.5% (LD, 4.8%; ED, 2.3%), and the 10-year survival rate was 1.8% (LD, 2.5%; ED, 1.2%) [2].

Few case reports have been published in the literature where patients with LD SCLC survived for years (5-11 years) before developing a relapse. Gressner, et al. reported a case of a patient with LD SCLC who survived for 10 years after receiving nine lines of chemotherapy. Their study emphasized the fact that repeated chemotherapy beyond second-line treatment in a subset of patients with LD SCLC may result in long-term survival, even without ever achieving a complete remission [3]. However, our patient, who only received four cycles of platinum-based chemotherapy along with TRT and whole brain radiation, not only survived for more than eleven years but also did not show any evidence of tumor recurrence until now.

There is an exceedingly rare number of cases reported in the literature where patients had disease-free survival for more than five years after receiving only the first line cisplatin and etoposide combination chemotherapy and TRT [4]. Matsui, et al. presented two cases where patients with LD survived for more than 10 years. In the first case, the patient had a surgical resection and received cisplatin-etoposide combination chemotherapy. The other patient was treated with cyclophosphamide, doxorubicin, and vincristine, alternating with cisplatin-etoposide for six cycles and was disease-free for 11.4 years [5]. Table 1 below summarizes a few of the case reports on long-term survivors of SCLC. 

**Table 1:** Long-term Survivors of SCLC OS: Overall survival; DFS: Disease-free survival

Case Reports	Number of Years Survived	Treatment Received
Long-term survival of a patient with small cell lung cancer after nine lines of chemotherapy and radiation	OS: 10	9 lines of chemotherapy, TRT, afterloading, and radionuclide therapy [3].
Long-term survival in small cell lung cancer: a case report and review of the literature	DFS: 7	Several lines of chemotherapy, radiotherapy, and long-acting somatostatin analogs [6].
Relapse of Stage I small cell lung cancer ten or more years after the start of treatment	Case 1: DFS: 10	Case 1: Lobectomy, 4 cycles of cisplatin-etoposide chemotherapy.
	Case 2: DFS: 11.4	Case 2: Cyclophosphamide, doxorubicin, and vincristine alternating with cisplatin-etoposide, for 6 cycles followed by TRT [5].
Long-term survival of a patient with small cell lung cancer associated with cancer-associated retinopathy	DFS: 9	4 cycles of cisplatin-etoposide along with TRT [4].
Ten years of disease-free survival between two diagnoses of small-cell lung cancer: a case report and a literature review	DFS: 10 OS: 11	3 cycles of chemotherapy with cyclophosphamide, doxorubicin, and vincristine followed by TRT. Documentation about whole brain radiation unavailable [7].
Redevelopment of small cell lung cancer after a long disease-free period: a case report	DFS: 10	Chemotherapy with cisplatin, etoposide, and doxorubicin along with radiotherapy [8].

It is possible that relapses as late as 11 years after treatment are, in fact, new primary SCLC tumors. Non-SCLC and other tobacco-related primary cancers tend to predominate in reports on second primary malignancies [9]. The study done by Lassen, et al. [2] showed that the second primary tumors (SPT) occurred more frequently than late relapses in five-year survivors and were the main cause of death (35.2%). Whether a late second SCLC diagnosis beyond the fifth year of follow-up is a second primary tumor or a late recurrence is difficult to determine. Two reasons among others underlie this difficulty. First, there are only a small number of long-term survivors who are available for observation and analysis. Second is the absence of a demonstrable precursor lesion for SCLC. The definition reported by Martini and Melamed has been used frequently; a new lung lesion is considered an SPT if it has a different histological type, if it appears in a different lobe or lung without the involvement of the lymphatics that are common to the original site, or if a disease-free interval of two years has passed. It is well known, however, that SCLC relapses after two years of remission, and it has a high tendency to metastasize.

## Conclusions

Although a diagnosis of SCLC portends a poor prognosis in most cases, one may expect long-term disease-free survival in certain exceptional responders to the conventional treatment. Thus, a small number of patients with SCLC who survive beyond five years must be followed up carefully as they are at a high risk of developing a relapse or secondary malignancies. Appropriate intervention must be performed as early as possible.
